# Diabetic Polyneuropathy in Type 1 and Type 2 Diabetes Mellitus: A Cross-Sectional Study

**DOI:** 10.7759/cureus.30004

**Published:** 2022-10-06

**Authors:** Emad Hindi, Bushra A Almusally, Rime Bawareth, Wala Alhamadah, Reem Alfaraj, Alhanouf Almwled, Aya Mousa, Manar Saga

**Affiliations:** 1 Anatomy, King Abdulaziz University Faculty of Medicine, Jeddah , SAU; 2 Internal Medicine, King Abdulaziz University Faculty of Medicine, Jeddah, SAU; 3 Medicine, King Abdulaziz University Faculty of Medicine, Jeddah, SAU

**Keywords:** diabetic polyneuropathy(dpn), diabetic complications, global physical activity questionnaire, michigan neuropathy screening instrument, physical activity, s: diabetes mellitus

## Abstract

Background: Diabetes mellitus (DM) is one of the three most common chronic diseases worldwide. This study aimed to determine the prevalence of diabetic polyneuropathy (DPN) among patients with diabetes.

Method: This cross-sectional study was carried out on DM patients who visited King Abdulaziz University Hospital (KAUH) between August 2021 and February 2022. We used the Michigan Neuropathy Screening Instrument (MNSI) questionnaire to determine if the patients had DN. In addition, we used the Global Physical Activity Questionnaire (GPAQ) to assess the level of physical activity (PA) in these DM patients.

Results: A total of 336 patients consented to participate in the study. We found a DN prevalence of 23.8% amongst DM patients treated at the KAUH. In addition, the prevalence of DN amongst T1DM and T2DM patients was found to be 16% and 24.4%, respectively. Furthermore, we found that 65% of DM patients developed complications, with a significant correlation observed between the duration of DM and the development of complications. However, patient age and sex were non-statistically significantly correlated with the development of complications. Analysis of the GPAQ showed that among the 249 patients who completed the questionnaire, none had a high physical activity level, while 4% and 96% had moderate and low physical activity levels, respectively. No association was found between physical activity and patients’ age, sex, type of DM, duration of DM, and development of complications.

Conclusion: DN prevalence amongst DM patients treated at KAUH was 23.8%. The duration of diabetes was found to be a risk factor for DN. However, patient age and sex were non-statistically significantly associated with DN.

## Introduction

Diabetes mellitus (DM), sometimes known as diabetes, is a chronic disease in which the body is unable to make or efficiently utilise the hormone insulin, resulting in elevated amounts of glucose in the blood [1]. DM comes in several forms, including gestational diabetes, type 1, type 2, and maturity-onset diabetes of the young. The most prevalent and well-known subtypes of DM continue to be type 1 DM (T1DM) and type 2 DM (T2DM). Juvenile diabetes or insulin-dependent diabetes, referred to as T1DM, is a multifactorial disease that mostly affects children and adolescents [1,2]. T2DM, on the other hand, typically affects adults due to environmental, genetic, and epigenetic aetiologies as well as a sedentary lifestyle and poor nutritional choices. Because of the distinctions in aetiology, each kind necessitates different management [2].

Insulin is a hormone that carries blood glucose to all of the body's cells, where it is converted to energy. This hormone is produced by the pancreatic beta cells [1-3]. Diabetes is characterised by hyperglycemia, or high blood glucose, which results from a lack of insulin or from cells' insensitivity to insulin [4]. Uncontrolled hyperglycemia can cause major consequences and serious life-threatening complications for patients, including cardiovascular disease, neuropathy, renal, and ocular diseases that can end in retinopathy and blindness. On the other hand, these serious problems can be delayed or avoided if diabetes is adequately treated [4].

Long-term diabetes can have many different complications, including diabetic neuropathy, which affects the peripheral nervous system as a whole and results in a sensory loss in the lower limbs [5]. Diabetic polyneuropathy (DPN) is a common illness that causes patients to fall more frequently and causes pain that is described as scorching or stabbing, numbness, hyperesthesia, or deep anguish. It is usually worst at night and affects the lower legs and feet, though it can also affect the hands in some people, lowering the quality of life (QOL) [6]. Sensory, motor, and autonomic neuropathies are the three forms of diabetic neuropathy. DPN, on the other hand, can lead to a variety of consequences, including persistent discomfort, foot ulcers, infections, and amputations [7].

A cross-sectional study carried out in Saudi Arabia used a questionnaire to gather the epidemiological and demographic data, as well as the medication histories, of 100 patients who attended the outpatient clinic for the treatment of DM. The prevalence of DPN was found to be 65.3% in DM patients [8]. Another cross-sectional study, which included 187 T2DM patients, was conducted at King Fahd University Hospital (KFUH) in the Al-Khobar district and found the prevalence of DPN to be 37.4% [9].

As only a few studies have addressed neuropathy as a complication of DM in Saudi Arabia, we carried out this study to determine the prevalence of DN among patients with diabetes treated at King Abdulaziz University Hospital (KAUH), Jeddah, Saudi Arabia.

## Materials and methods

This cross-sectional study was conducted from August 2021 to February 2022 at KAUH, a tertiary centre in Jeddah, Saudi Arabia, using pre-designed questionnaires. It was conducted under the department of medicine and approved by the Research Ethical Committee of KAUH with the following reference number 336-2. A total of 345 patients were recruited from the outpatient clinics, adult in-patient department, emergency department, and cardiac catheterization unit of the hospital. Included in this study were patients of both sexes aged 18 years and above with T1DM or T2DM. Patients with other types of diabetes, glucose intolerance, and gestational diabetes, as well as those aged below 18 years, were excluded from this study. Patients with a known history of neurological or neuropsychiatric disorders were also excluded. A total of nine patients were excluded due to a history of neurological or psychiatric disease, and finally, 336 patients were retained as the study participants.

A Google form application was used for questionnaire creation. Verbal consent was obtained from all the study participants. The questionnaire consisted of four sections. The first three sections were mandatory, while the fourth was optional. The first section collected information on the name of the data collector and the personal information of the participants, such as Medical Record Number (MRN), sex, age, and past medical history of any neuropsychiatric diseases, with the possibility of answering with a 'Yes' or a 'No'. Only participants who responded with a 'No' were included in the study. The other questions in this section were multiple choice questions that interrogated the type of DM, with only two choices available, i.e., T1DM or T2DM; the type of medication used by the participants, with five different choices, i.e., diet, hypoglycaemic medications, insulin, both hypoglycaemic medications and insulin, and other (which had to be mentioned), and the complications due to DM, with 'Yes' or 'No' as options; if the response here was 'Yes', the complication had to be mentioned. The Michigan Neuropathy Screening Instrument (MNSI) was used to evaluate DN in DM patients in the second and third sections of the questionnaire. The Arabic version of the MNSI was used and a total score of four or more was considered abnormal [10-12]. MNSI questions on medical history had to be answered with a 'Yes' or a 'No'. The MNSI questions on medical history and physical examination are listed in Table 1. The MNSI examination was performed by medical students under the supervision of a specialist, with a possible total score of eight points. A score of 2.5 or higher was considered abnormal [13].

**Table 1:** History and physical examination sections of the MNSI MNSI: Michigan Neuropathy Screening Instrument

MNSI: History Section	MNSI: Physical Examination Section
Are your legs and/or feet numb?	Right foot
Have you ever had any burning pain in your legs and/or feet?	1. Appearance of foot
Are your feet too sensitive to touch?	2. Ulcerations
Do you get muscle cramps in your legs and/or feet?	3. Ankle reflex
Have you ever had any prickling feelings in your legs or feet?	4. vibration perception at the great toe
Does it hurt when the bed cover touches your skin?	Left foot
When you get into the tub or shower, are you able to tell if the water is hot or cold?	1. Appearance of foot
Have you ever had an open sore on your foot?	2. Ulcerations
Has your doctor ever told you that you have diabetic neuropathy?	3. Ankle reflex
Do you have general weakness most of the time?	4. Vibration perception at great toe
Are your symptoms worse at night?	
Do your legs hurt when you walk?	
Are you able to sense your feet when you walk?	
Is the skin on your feet so dry that it cracks open?	
Have you ever had an amputation?	

The Arabic version of the Global Physical Activity Questionnaire (GPAQ) was used to assess physical activity (PA) in the fourth section of the questionnaire. The GPAQ was created by the WHO for country-level physical activity surveillance. It contains 16 questions that gather information on physical activity involvement in three categories and sedentary behaviours. Work activity, and recreational activities, were the three domains contained in the questionnaire. We categorised participants into the 'high', 'moderate', or 'low' groups based on their total and median PA. A total of 249 patients accepted to participate in the GPAQ section of the questionnaire [14].

The Statistical Package for the Social Sciences (SPSS; IBM Corp., Armonk, NY) software version 26 was used to perform statistical analyses on the collected data. The Chi-squared test (2) was used to analyse the association between qualitative and quantitative variables with their percentages. Quantitative data were presented as mean ± standard deviation (SD) and the Mann-Whitney U test was used to assess the relationship between quantitative variables. Spearman’s correlation analysis was carried out to compare non-parametric variables. Values of p<0.05 were considered statistically significant.

## Results

The mean age of the study participants was 55.32 ± 15.21 years, and 59.2% of them were female. The majority of them had T2DM (92.9%). The mean DM duration was 13.99 ± 9.35 years. Table 2 shows the variable matchings of the study participants. Most of the patients were on oral hypoglycaemic medications (45.5%); in addition, 28% of them were on oral hypoglycaemic medications in combination with insulin, and 25% of them were on insulin only. Of all the patients evaluated, 43.2% were found to have had complications such as retinopathy (18.2%), neuropathy (5.1%), and amputations (3.3%). The analysis included all the participants, and their mean MNSI score was 4.8 ± 2.72.

**Table 2:** Distribution of the study participants according to sex, diabetes type, medication used, and diabetic complications (no.: 336)

Variables		No. (%)
Sex	Female	199 (59.2)
	Male	137 (40.8)
Type of DM	T1DM	24 (71)
	T2DM	213 (92.9)
DM treatment strategy	Diet	3 (0.9)
	Nothing (others)	2 (0.6)
	Oral hypoglycaemic medications	153 (45.5)
	Oral hypoglycaemic medications and insulin	94 (28)
	Insulin	84 (25)
DM complications	No	191 (56.8)
	Yes	145 (43.2)
If yes, what complications? (No.:145; more than one answer was allowed)	Stroke	7 (2.1)
	Amputation	11 (3.3)
	Renal failure	1 (0.3)
	Cataract	31 (9.2)
	Retinopathy	61 (18.2)
	Nephropathy	32 (9.5)
	Neuropathy	17 (5.1)
	Cardiovascular diseases	17 (5.1)
	Glaucoma	12 (3.6)
	Gangrene	4 (1.2)
	Diabetic foot	6 (1.8)

**Figure 1:**
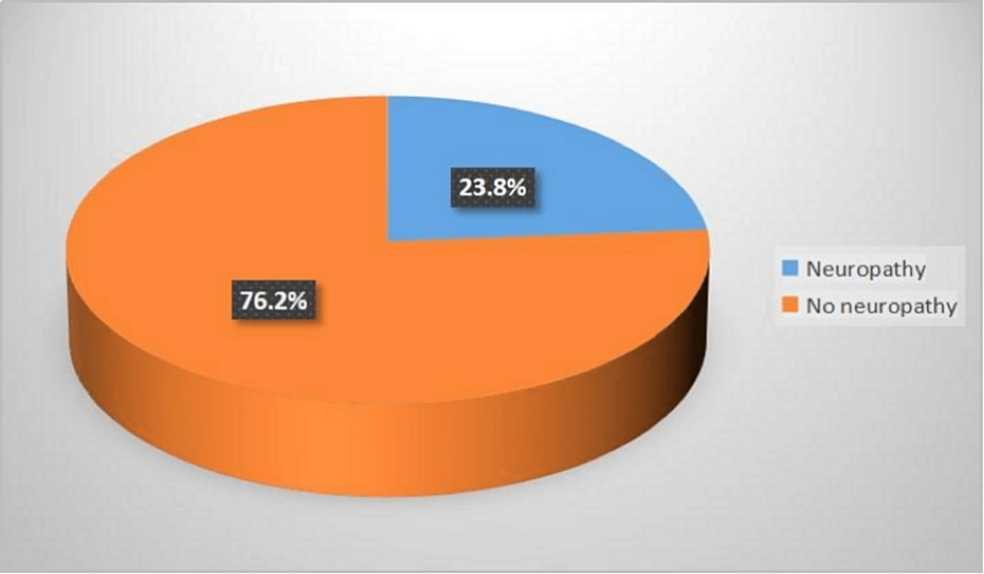
Prevalence of diabetic neuropathy based on the MNSI classification MNSI: Michigan Neuropathy Screening Instrument

**Figure 2:**
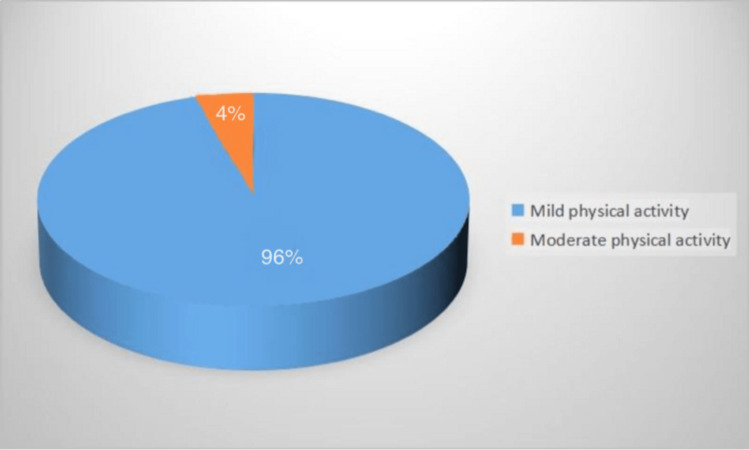
Physical activity levels among the study participants (no.:249)

As illustrated in Figure 1, based on the MNSI classification, 80 patients (23.8%) were found to have had DN while 256 (76.2%) patients did not have DN. As shown in Figure 2, among the 249 patients who completed the PA questionnaire, none had a high PA level, while 4% and 96% had moderate and mild PA levels, respectively.

As indicated in Table 3, no significant difference in age was observed between patients with DN and those without DN, as the mean age in patients with DN was 55.08 ± 16.14 and that in patients without DN was 55.4 ± 14.94. Among the patients who had DN, 53.75% and 46.25% were female and male, respectively, and among those without DN, 60.94% and 39.06% were female and male, respectively. Moreover, diabetes type was associated with DN, as 5% of participants with T1DM had DN and 95% of them did not have DN. As concerns patients with T2DM, 92.19% had DN and 7.8% did not have DN. We also found a significant association between the duration of DM and the occurrence of DN, as patients with DN had an average DM duration of 17.7 ± 9.42 years, while patients without DN had an average DM duration of 12.82 ± 9.04 years. Amongst patients with DN, 8.75% had amputations, 25% had retinopathy, and 3.75% had diabetic foot. However, among patients without DN, 1.56% had amputations, 14.06% had retinopathy, and 1.17% suffered from diabetic foot.

**Table 3:** Relationship between diabetic neuropathy prevalence and patient age, sex, diabetes type, diabetes duration, and type of complication (no.: 336)

Variable		DN no. (%)	No DN no. (%)	p-value
Age		54.4 ± 16.05	55.61 ± 14.96	0.532
Sex	Female	43 (53.75)	156 (60.94)	0.253
	Male	37 (46.25)	100 (39.06)	
Type of DM	T1DM	4 (5)	76 (95)	0.394
	T2DM	236 (92.19)	20 (7.81)	
Diabetes duration		17.5 ± 9.15	12.88 ± 9.16	<0.001
Diabetes complications (more than one answer was allowed)	Stroke	2 (2.5)	5 (1.95)	0.765
	Amputation	7 (8.75)	4 (1.56)	0.002
	Cataract	5 (6.25)	26 (10.16)	0.292
	Retinopathy	25 (25)	36 (14.06)	<0.001
	Nephropathy	15 (18.75)	17 (6.64)	0.001
	Cardiovascular diseases	7 (8.75)	10 (3.91)	0.084
	Glaucoma	2 (2.5)	10 (3.91)	0.554
	Gangrene	3 (3.75)	1 (0.39)	0.016
	Diabetic foot	3 (3.75)	3 (1.17)	0.129

**Figure 3:**
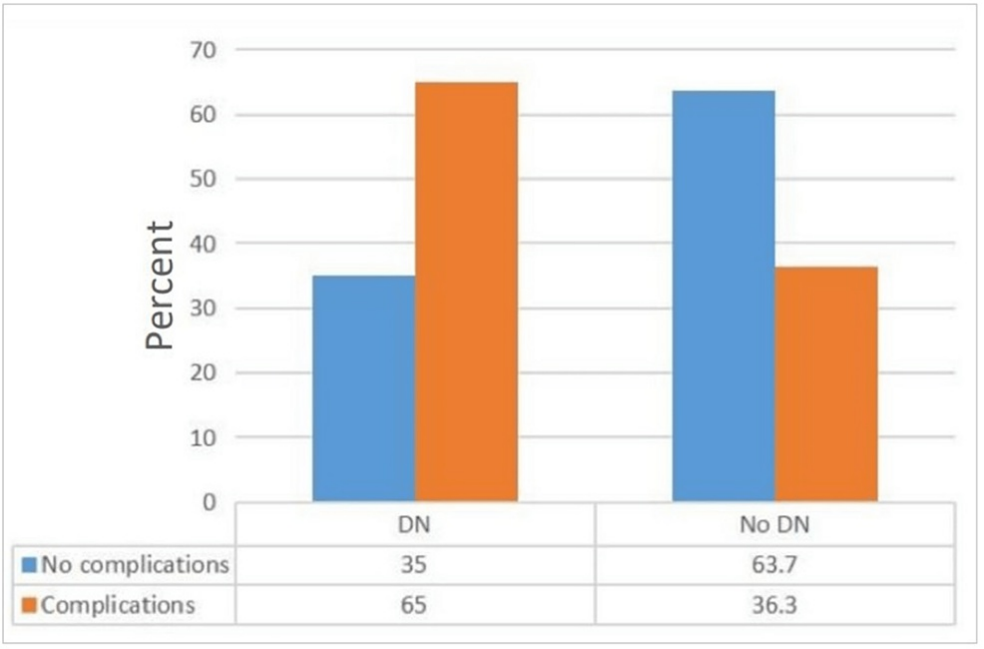
Relationship between diabetic neuropathy prevalence and the presence of complications (no.: 336) The figure shows the relationship between diabetic complications and the development of diabetic neuropathy. The Y-axis represents the percentage of patients with or without complications. N.B.: (χ^2^ = 20.42; p-value ≤ 0.001).

We evaluated the relationship between DN and the development of other diabetic complications and found that 65% of DN patients had other diabetic complications, while only 35% of them did not have other complications. Only 36.3% of patients without DN developed diabetic complications, while 63.7% of them did not develop any diabetes-related complications (Figure 3).

**Figure 4:**
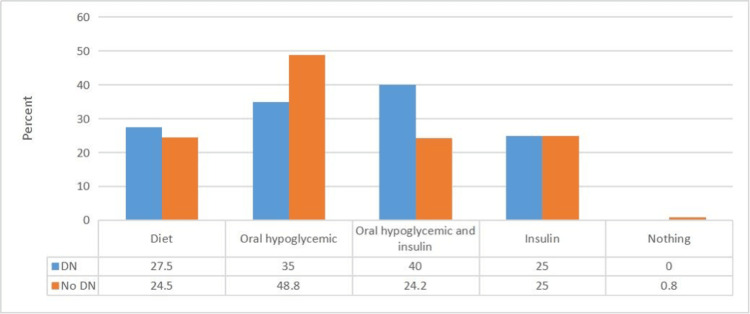
Relationship between the presence or absence of diabetic neuropathy and diabetes mellitus treatment strategy used (no.: 336) N.B.: (χ^2^ = 9.45; p-value = 0.049).

As shown in Figure 4, DN patients were on different management plans, including diet modifications, oral hypoglycaemic agents, oral hypoglycaemic agents and insulin, and insulin only in 27.5%, 35%, 25%, and 40% of them, respectively. As concerns patients without DN, 24.5%, 48.8%, 24.2%, and 25% of them were on diet modification, oral hypoglycaemic agents, oral hypoglycaemic agents and insulin, and insulin only, respectively.

**Figure 5:**
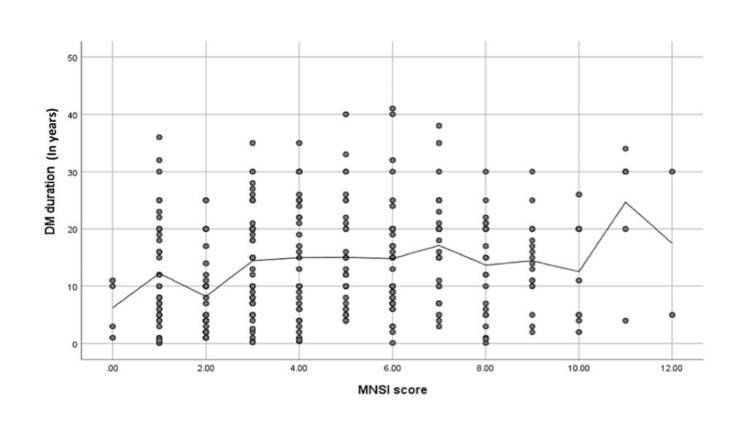
Spearman's correlation analysis between MNSI scores and diabetes mellitus duration N.B.: (r = 0.19; p-value ≤ 0.001) MNSI: Michigan Neuropathy Screening Instrument.

As shown in Figure 5, we found a highly positive and significant correlation between MNSI score and DM duration (Spearman’s r = 0.19, p-value ≤ 0.001). The cut-off for DN scores was 7.0 or more. The overall median time for DN development was found to be 15 years.

The relationship between patient PA level as determined using the GPAQ and patient age, sex, diabetes type, DM duration, and DM complications was evaluated. We found that the mean age for patients who practised mild PA was 57.3 ± 13.05 years. Furthermore, the mean age for patients who practised moderate PA was 62.82 ± 8.1 years. 96% of patients with DM practised mild PA, while 4% practised moderate PA. Diabetes duration in patients who enrolled in mild PA ranged from 4.69 to 9.6 years and 10.11 to 10.41 years in patients who enrolled in moderate PA. In Table 4, the relationship between PA and patient sex and DM complications is shown. The majority of patients were female (54.6%), as 45.4% of them were male. We also found that 45% of patients who practise mild and/or moderate PA develop complications, while 55% of them do not develop any complications.

**Table 4:** Relationship between patient physical activity level and patient sex, diabetes type, and the development of complications (no.: 249)

Variable		Mild physical activity no. (%)	Moderate physical activity no. (%)	p-value
Sex	Female	130 (52.2)	6 (2.4)	0.996
	Male	37 (46.25)	100 (39.06)	
Type of DM	T1DM and T2DM	239(96)	9.96(4)	0.39
Diabetes complications	Yes	130 (52.2)	7 (2.8)	0.557
	No	108 (43.4)	4 (1.6)	
Diabetes complications (more than one answer was allowed)	Stroke	4 (3.9)	0 (0.0)	0.665
	Amputation	7 (6.7)	0 (0.0)	0.564
	Renal failure	28 (26.9)	0 (0.0	0.227
	Cataract	4 (3.8)	3 (2.8)	0.369
	Retinopathy	24 (23.1)	1 (0.9)	0.915
	Nephropathy	17 (16.3)	0 (0.0)	0.358
	Cardiovascular diseases	11 (10.6)	0 (0.0)	0.466
	Glaucoma	5 (5)	0 (0.0)	0.62

## Discussion

The global prevalence of DM was 366 million in 2011 and it was predicted that this number would reach 552 million by 2030, indicating a 7.7% increase in prevalence [5]. According to WHO statistics, Saudi Arabia is the second and seventh country in the Middle East and the world, respectively, with the highest DM prevalence [15]. Due to the increasing number of diabetes cases in the country over the years [16], we evaluated the prevalence of DN in DM patients, as it is one of the most common complications of long-term DM [17]. In this study, the MNSI was used to estimate the prevalence of DN in both T1DM and T2DM patients. We found the prevalence of DN in T1DM and T2DM patients to be 16% and 24.4%, respectively. In addition, the estimated prevalence of DN in both T1DM and T2DM patients was 23.8%. Furthermore, 65% of patients who developed diabetic complications had DN. A cross-sectional study carried out in October 2020 on DN in Arar, Northern Saudi Arabia, found its prevalence in 208 DM patients to be 26.4% [18].

Figure 5 shows the MNSI scores of the patients, as well as the relationship between DN and diabetes duration; a significant positive association was found between DN and diabetes duration. This score was found to increase with an increase in the duration of DM. Based on our findings, the time it takes to develop DN depends on how long the patient has had DM, and this could range from 8 to 16 years. A similar cross-sectional study was conducted in Egypt in 2020 at the Beni-Suef University Hospital and included patients with DM and DN who fulfilled the American Diabetes Association (ADA) criteria. A total of 25 patients were evaluated using the MNSI and it was found that 50% of diabetics acquire neuropathy between 25 and 30 years following diagnosis [19]. Another cross-sectional study carried out in Saudi Arabia on a sample size of 552 patients with an average age of 53.4 years, published in 2014, showed that the incidence of DN was 12.19-fold higher in patients who had diabetes for 20 or more years than in those who had diabetes for 2-5 years [20].

As shown in Figure 4, the rate of use of hypoglycaemic agents in combination with insulin was higher in patients with DN than in those without DN, indicating that there exists an association between poor diabetes control and DN. Similarly, in another cross-sectional study carried out in Saudi Arabia in 2014, which included 552 patients with diabetes, insulin use was found to be associated with DN [21]. Another cross-sectional study carried out in Qassim, Saudi Arabia in 2020 on 374 patients with diabetes showed that a multi-drug approach and insulin use, in particular, was associated with a high risk of developing DN [11].

We found a significant positive correlation between the occurrence of diabetic complications and the development of DN. These complications usually include cardiovascular diseases, such as myocardial infarction, nephropathy, and lower extremity amputation [22]. In the Western world, diabetes is the leading cause of neuropathy [23]. In the context of our study, the presence of diabetic complications would most likely indicate the simultaneous existence of DN. As illustrated in Figure 3, DN would most likely occur in patients with diabetes prior to the development of diabetic complications. DN accounts for higher hospitalisation rates than other diabetic complications [24]. A case-control study carried out in Pakistan in 2015, which evaluated the association between DN and glycaemic control and diabetes duration, demonstrated the existence of a correlation between DN and several complications. Nisar et al. reported that early screening and detection would aid in delaying the progression of neuropathy and minimise the risk of developing diabetic complications [23].

Our findings showed that there are no significant sex-based differences among DN patients. These findings are similar to those of other studies. A retrospective, nested case-control study conducted in Riyadh, Saudi Arabia in 2018 on 2906 patients showed an insignificant association between sex and DN [25]. In addition, a retrospective cross-sectional study carried out in Lahore, Pakistan, and published in 2014 showed that there was no association between sex and DN [26]. However, many internationally published studies have evaluated sex-based differences in patients with DN. Actually, a prospective, cross-sectional, multicentre study carried out in eight hospitals in Italy demonstrated the existence of an association between neuropathic pain and sex [27]. We evaluated the association between DN prevalence and patient age, sex, diabetes type, diabetes duration, and type of diabetic complication, and found that there was no association between age and DN (Table 3).

Habitual PA was found not to be associated with patient age, sex, diabetes duration, or diabetic complications (Table 4). However, they found that 45% of patients who practise mild and/or moderate PA develop complications, while 55% of them do not develop any complications. Another study carried out on 339 patients with diabetes aged 40-85 years as part of the 2003-2004 National Health and Nutrition Examination Survey, showed that proper PA in combination with good glycaemic control reduces the risk of neuropathy [28].

Limitations of the study

Our study had some limitations. First, the gold standard test for diagnosing DPN, which is a nerve conduction study, was not performed, leaving the result of the study probable but not definite. Second, reflex examinations for our questionnaire were examiner-dependent, with a high error margin. The third limitation was that our sample size was relatively small. The majority of the patients were T2DM, making the sample size of patients with T1DM even smaller. Another limitation encountered due to the nature of the study, as it required a detailed medical history and physical examination, was that some patients found it time-consuming to participate.

## Conclusions

In this study, DPN prevalence amongst DM patients treated at KAUH was 23.8%. We also found a significant association between the duration of DM and the development of DPN. As such, patients with DPN had an average duration of 17.7 ± 9.42 years. Moreover, we evaluated the relationship between DPN and the development of other DM-related complications and found that 65% of patients with DPN had other DM-related complications. Patients with DM in this study had different treatment strategies, including diet, oral hypoglycemic medications, insulin, and others. We found that the rate of using hypoglycemic agents in combination with insulin was higher in patients with DPN than in those without DPN. Patient sex and age had no significant relationship with the development of DPN.
